# Comparative efficacy of dental implant motor–assisted versus conventional temporomandibular joint arthrocentesis for disc displacement with reduction (a randomized controlled clinical trial)

**DOI:** 10.1186/s12903-026-08334-2

**Published:** 2026-04-25

**Authors:** Ahmed Saied Mohamed, Ragab Shaaban, Gaafar Nabil El Halawani, Aya A. Sakr

**Affiliations:** Oral and Maxillofacial Surgery Department, Faculty of Dentistry, Alexandria University, Alexandria, Egypt

**Keywords:** Temporomandibular disorders, Arthrocentesis, Implant motor, Disc displacement with reduction, Minimally invasive therapy

## Abstract

**Background:**

Temporomandibular joint internal derangement (TMJID), particularly disc displacement with reduction (DDwR), represents a common subtype of temporomandibular disorders (TMDs). Arthrocentesis is considered a minimally invasive option for selected patients with persistent symptoms. Standardization of irrigation delivery may improve procedural efficiency and reproducibility.

**Aim:**

To compare TMJ arthrocentesis performed using a dental implant motor–assisted irrigation system at two different power settings with the conventional manual technique in patients with DDwR, primarily in terms of maximum mouth opening (MMO), and secondarily regarding pain intensity, mandibular movements, and procedure duration.

**Materials and methods:**

A prospective randomized controlled parallel-group clinical trial was conducted on 30 patients diagnosed with DDwR. Participants were randomly allocated into three equal groups (*n* = 10 each). Group A received motor-assisted arthrocentesis at 80% power, Group B at 40% power, and Group C underwent conventional syringe-based arthrocentesis. Clinical evaluation included MMO (primary outcome), pain intensity, mandibular movements, and procedure duration at 1 week and 3 months postoperatively.

**Results:**

All groups demonstrated significant improvement in pain and mandibular movement parameters over time (*p* < 0.05). MMO increased significantly in all groups, with Group A showing greater improvement at 3 months (41.6 ± 1.26 mm) compared with Group B (39.0 ± 1.33 mm) and Group C (39.7 ± 1.25 mm) (*p* = 0.016). Procedure duration differed significantly among groups (*p* = 0.001), with Group A demonstrating the shortest mean operative time (14.9 ± 2.33 min). No major complications were observed; minor transient swelling occurred in both techniques.

**Conclusions:**

Both conventional and motor-assisted TMJ arthrocentesis resulted in significant functional improvement in patients with DDwR. The motor-assisted technique demonstrated shorter operative time; however, due to the limited sample size, these findings should be interpreted with caution and cannot be considered definitive. Larger, well-powered studies are warranted to confirm these findings.

**Trial registration:**

The study was registered at ClinicalTrials.gov under the identifier NCT07239037. Participant recruitment was conducted between October 2024 and July 2025 at the Outpatient Clinic of the Oral and Maxillofacial Surgery Department, Faculty of Dentistry, Alexandria University, Egypt. Registered was on 16/11/25, retrospectively registered. The delay in registry submission was related to administrative and procedural factors during the finalization of the trial documentation. Importantly, the study protocol, eligibility criteria, outcome measures, and statistical analysis plan were defined a priori and were not modified after recruitment or data collection had commenced. The trial protocol and statistical analysis plan are publicly accessible through the registry.

**Supplementary Information:**

The online version contains supplementary material available at 10.1186/s12903-026-08334-2.

## Introduction

Temporomandibular joint disorders (TMDs) represent a heterogeneous group of conditions affecting the temporomandibular joint (TMJ) and associated structures, encompassing a wide range of clinical presentations and symptoms [1, 2]. The prevalence of TMDs is estimated to be substantial, affecting millions of individuals worldwide [3]. 

Internal derangements of the temporomandibular joint (TMJ) are conditions in which the articular disc has become displaced from its normal relationship with the mandibular condyle and glenoid fossa [4]. The displacement can manifest in several forms, most commonly as disc displacement with reduction (where the disc returns to its physiological position during mandibular opening, sometimes accompanied by clicking or popping) or disc displacement without reduction (where the disc remains displaced during opening, possibly resulting in restricted mouth opening) [5]. 

Temporomandibular joint disorders (TMDs) are a group of conditions involving the temporomandibular joint, masticatory muscles, and associated structures, with a well-established multifactorial etiology. Current evidence indicates that the development and persistence of TMDs are influenced by an interplay of biological, biomechanical, and psychosocial factors, including parafunctional habits, trauma, central pain modulation mechanisms, and psychological stress. Occlusal factors and various forms of malocclusion have also been proposed as potential contributing or perpetuating elements; however, their precise role remains controversial, and a direct causal relationship has not been conclusively established. As a result, contemporary concepts of TMD management emphasize conservative, minimally invasive, and symptom-oriented approaches that aim to restore joint function and alleviate pain rather than focusing on correction of occlusal discrepancies alone. This multifaceted understanding has led to the adoption of multidisciplinary treatment strategies tailored to individual patient presentations [6–9]. 

The link between malocclusion and TMD is particularly contentious. An unstable occlusion might disrupt TMJ load-bearing capacity, possibly contributing to TMD development. While tooth-clenching and grinding are acknowledged as significant factors, the exact contribution of malocclusion remains debated [10]. 

A comprehensive treatment strategy for TMDs typically encompasses a spectrum of non-surgical and surgical modalities [11]. 

A nonsurgical approach is generally recommended as the initial management for temporomandibular disorders (TMDs), with surgical intervention reserved for cases unresponsive to conservative therapies [12]. 

Conservative treatment methods for TMDs: pharmacotherapy, physical therapy, inter-occlusal splints, hyaluronic acid (HA) joint injection, botulinum toxin (BTX) type A injection, arthrocentesis, and arthroscopic treatment [12]. 

Over recent years, arthrocentesis of the TMJ has emerged as a minimally invasive and effective technique for the management of TMDs. Arthrocentesis is commonly defined as the lavage of the superior joint compartment using sterile needles and irrigating solutions, without direct visualization of the joint space [13]. The procedure aims to alleviate pain by removing intra-articular inflammatory mediators and to improve mandibular mobility by disrupting adhesions through hydraulic pressure generated by the irrigation fluid [14]. 

Beyond lavage, many studies report intra‑articular injections of substances like hyaluronic acid (HA), corticosteroids, PRP, PRF, and others to enhance outcomes following arthrocentesis [15]. 

Recently, the use of a dental implant motor irrigation pump for arthrocentesis has been introduced as a novel modification of the conventional technique. However, to date, only a single study has reported on this approach [16], highlighting the limited evidence available. Therefore, the present study aims to compare the clinical outcomes of TMJ arthrocentesis performed using a dental implant motor irrigation pump using two different irrigation powers, with those of the conventional technique.

The purpose is to evaluate whether employing a dental implant motor irrigation pump during TMJ arthrocentesis offers measurable clinical advantages over the conventional technique.

The hypothesis of this study was that there would be significant difference between TMJ arthrocentesis using an implant motor irrigation pump and the conventional arthrocentesis technique in the management of TMJID.

This study aims to compare the clinical outcomes including pain, mandibular movements and procedure duration of TMJ arthrocentesis performed using a dental implant motor irrigation pump at two different power settings (80% and 40%) with those of the traditional arthrocentesis technique in the management of TMJID.

## Materials and methods

**Fig. 1:**
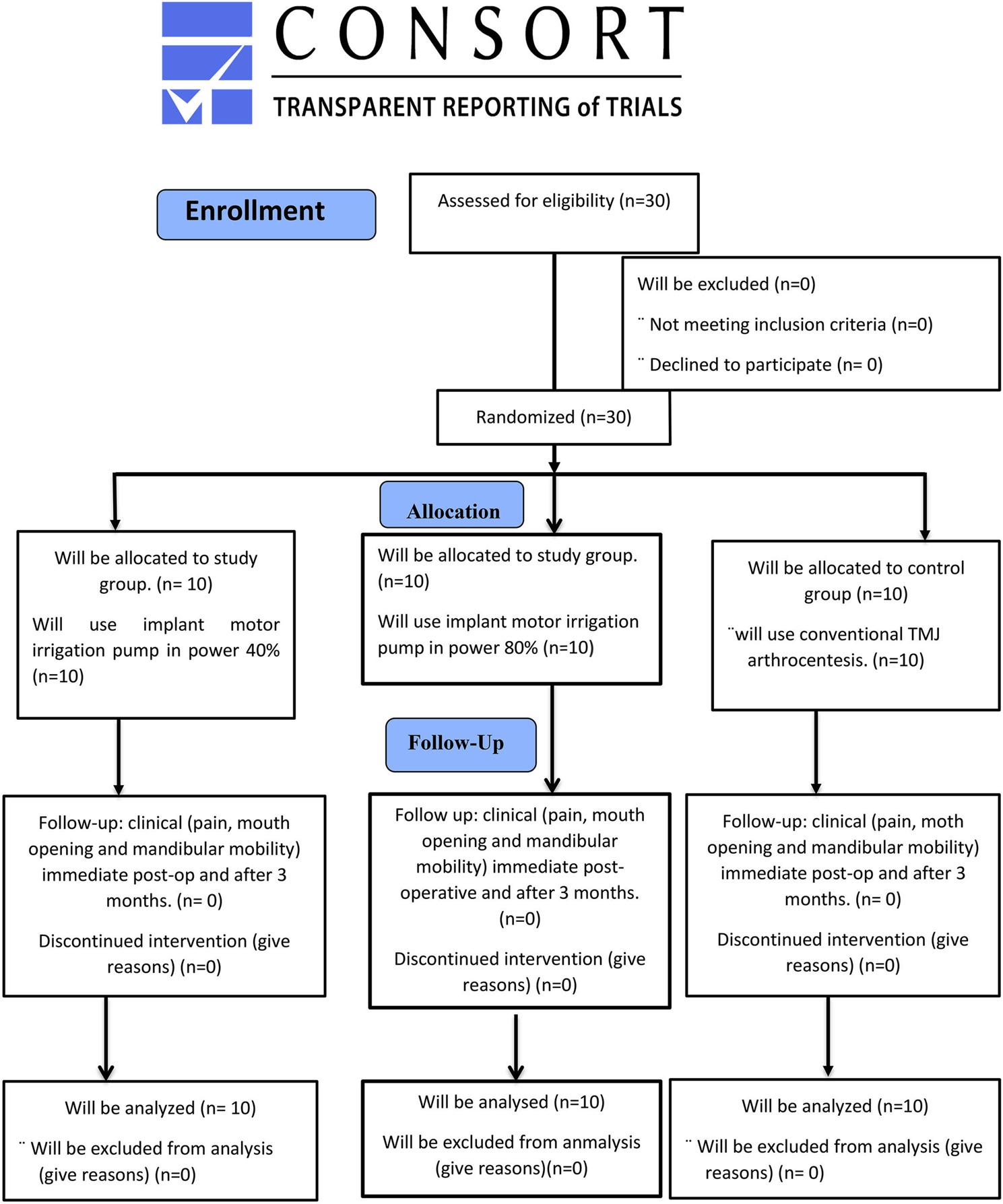
CONSORT flow diagram of patient recruitment, allocation, follow-up, and analysis

The study was a prospective parallel, exploratory, randomized controlled clinical trial that was designed and reported in accordance with the CONSORT guidelines [17]. (Fig. 1). Conducted in accordance with the principles of the World Medical Association Declaration of Helsinki (2013).

Patients were randomly allocated into three groups according to the principles of a randomized controlled clinical trial and the assigned treatment modality. Block randomization with a block size of four was performed for the three groups using a computer-generated random number list (https://www.randomizer.org). The random sequence was generated by an independent statistician who was not involved in patient recruitment or outcome assessment. Allocation concealment was ensured using sealed, opaque, sequentially numbered envelopes prepared by the statistician.

Regarding assessment of MMO, pain intensity, mandibular movements, and procedure time, assessor blinding was not feasible, as measurements were performed by two members of the research team. Therefore, all measurements were recorded by two calibrated assessors with repeated measurements who demonstrated excellent intra and inter-examiner reliability, as indicated by very high ICC values (≥ 0.99) with narrow confidence intervals close to 1.0 and all data were recorded prospectively to mitigate any potential bias.

Diagnosis of disc displacement with reduction (DDwR) was established through a standardized clinical examination combined with magnetic resonance imaging (MRI) confirmation. Clinical assessment included detection of reproducible joint clicking during mandibular opening and closing, absence of persistent locking, and evaluation of mandibular range of motion. MRI was performed to confirm anterior disc displacement with reduction during dynamic jaw movement. MRI examinations were performed using a 1.5-Tesla scanner with dedicated TMJ surface coils. Imaging was obtained in both closed- and open-mouth positions using sagittal and coronal sections oriented perpendicular and parallel to the long axis of the mandibular condyle. T1-weighted and proton density–weighted sequences were used to evaluate disc position and joint structures. Disc displacement with reduction was diagnosed when anterior displacement of the disc was observed in the closed-mouth position with recapture of the disc upon mouth opening. The MRI diagnostic approach was guided by contemporary TMJ imaging standards as described in recent literature [18, 19]. 

All MRI scans were interpreted by an experienced radiologist according to predefined radiologic criteria aligned with contemporary TMJ MRI standards to ensure diagnostic consistency.

All examinations were performed by calibrated oral and maxillofacial surgeons using a standardized clinical protocol to ensure diagnostic consistency. Calibration sessions were conducted prior to study initiation to standardize clinical interpretation of joint sounds, mandibular movement patterns, and MRI findings. The DC/TMD protocol was not formally administered as a structured diagnostic instrument; therefore, Axis II psychosocial assessment was not included in the present study.

Participants presenting for the management of DDWR were prospectively recruited between October 2024 and July 2025 at the Outpatient Clinic of the Oral and Maxillofacial Surgery Department, Faculty of Dentistry, Alexandria University, Egypt. Eligible patients included individuals of both genders, aged 15–50 years.

Patients with a history of TMJ surgery, those with relevant medical comorbidities, and individuals presenting with overlying infection or cellulitis in the TMJ region were excluded from the study.

Patients were clinically evaluated for multiple parameters. The primary outcome was maximal mouth opening (MMO), measured in millimeters. Secondary outcomes included pain intensity, assessed using a visual analog scale (VAS); mandibular movements, including lateral excursions and protrusion, measured in millimeters; and procedure duration, recorded in minutes, was measured from the initiation of local anesthesia administration until completion of the irrigation procedure and needle removal. All assessments were performed at 1-week, 4-weeks, and 3-month follow-up intervals.

Maximum mouth opening (MMO) was measured as the interincisal distance between the incisal edge of the maxillary central incisor and the corresponding mandibular central incisor using a calibrated digital caliper. In cases of minor incisal wear or discrepancy, consistent anatomical reference points were maintained across all follow-up visits. Lateral excursions and protrusive movements were measured from the midline of the maxillary central incisors to the midline of the mandibular central incisors during maximal movement. All measurements were performed with the patient in an upright position and repeated twice, with the mean value recorded.

Occlusal classification (Angle class) was not used as an inclusion or exclusion criterion in the present study. Patients with severe malocclusion that could interfere with reliable interincisal measurement were not encountered during recruitment.

Ethical approval was guaranteed by the ethics committee, IRB by OHRP: 00010556. IORG: 0008839. (Approval No: 0911-05/2024).

### Study design and group allocation

Eligible patients were randomly allocated into three equal groups (*n* = 10 per group) using computer-generated random numbers to ensure unbiased distribution:

Group A (Study Group 1) received TMJ arthrocentesis performed using a dental implant motor irrigation pump operating at 80% power, while Group B (Study Group2) underwent the same technique but with the pump operating at 40% power. Group C (Control Group) served as the control group and was treated using the conventional TMJ arthrocentesis technique.

### Preoperative phase

#### Splint therapy

All patients received anterior repositioning splint therapy prior to arthrocentesis, in accordance with evidence suggesting that the combination of splint therapy and arthrocentesis provides superior clinical outcomes in the management of temporomandibular joint internal derangement.

To minimize potential confounding effects and ensure consistency across study groups, anterior repositioning splint therapy was standardized and prescribed to all participants for a total duration of three months, including the postoperative follow-up period. Patients were instructed to wear the splint continuously as part of the treatment protocol.

### Procedure

**Fig. 2:**
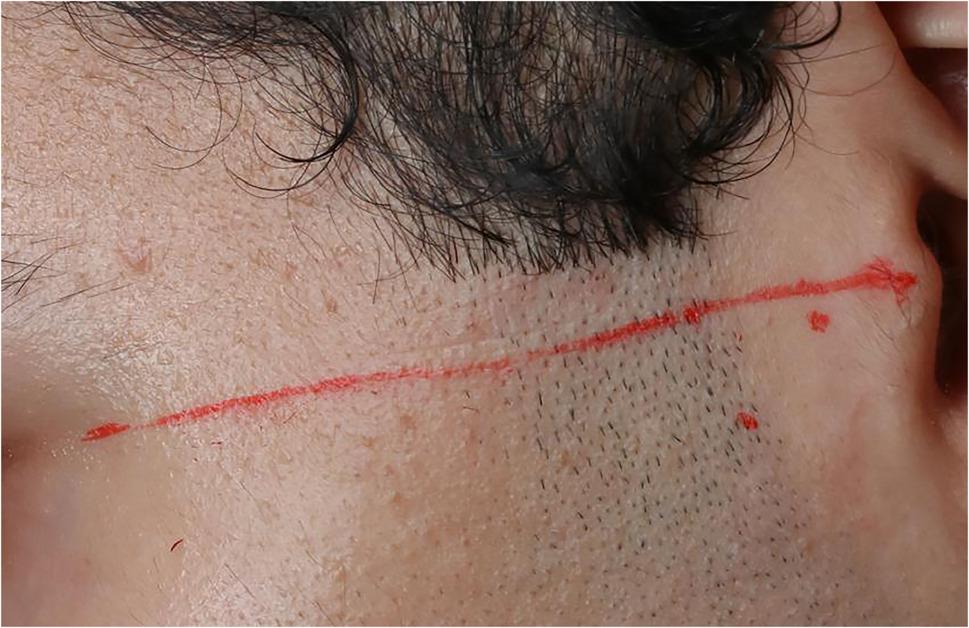
Drawing of cantho-tragus line

A surgical site marker was used to draw a straight line from the midpoint of the tragus to the lateral canthus of the eye (cantho-tragus line). Two points for needle insertion were marked along this line: the first, 10 mm anterior to the tragus and 2 mm below the line; the second, 20 mm anterior to the tragus and 10 mm inferior to the line. All measurements were obtained using a digital caliper to ensure accuracy. Local infiltration anesthesia was administered using 1.8 mL of Artinibsa 4% with 1:100,000 epinephrine. (Fig. 2)

With the patient’s mouth maximally opened to displace the condyle downward and forward, the first 18 gauge needle (40 mm × 1.2 mm) was introduced into the posterior recess of the upper compartment. The needle was directed anteriorly, superiorly, and medially until its tip contacted the mandibular fossa. The needle was connected to a 5-mL syringe, and 4 mL of 0.9% saline was injected to distend the joint space. A second needle of the same dimensions was then introduced at the pre-marked entry point and connected to a 60-mL syringe.

### Dental implant motor irrigation pump technique (study group)

**Fig. 3:**
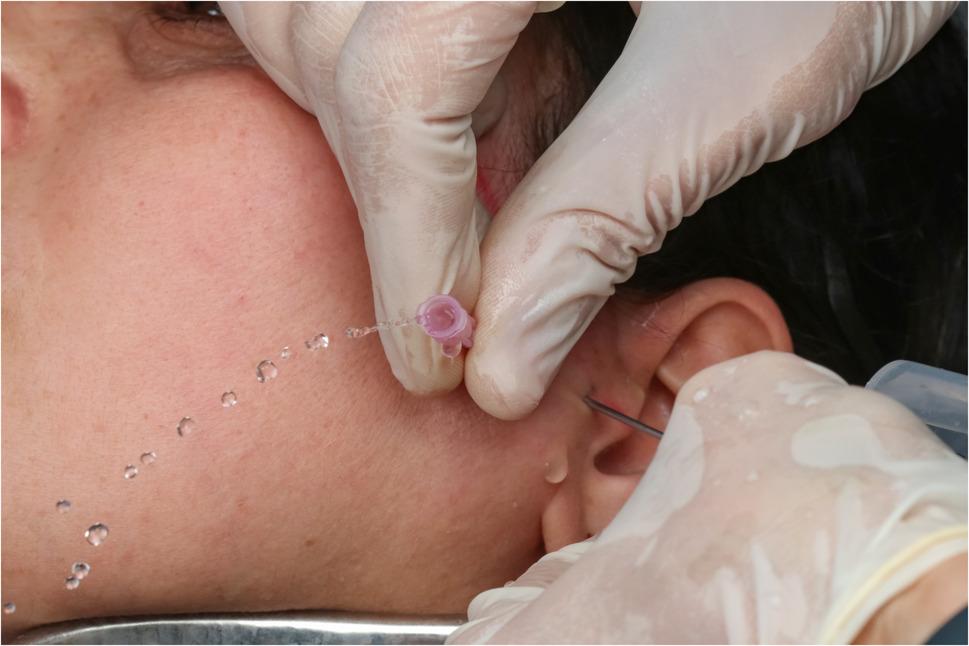
Implant motor in 80% irrigation power

A plastic extender was attached to the first needle, which was then connected to the dental implant motor irrigation pump. A total of 250 mL of 0.9% saline solution was delivered via the pump. In Study Group 1, the pump was set at 80% power, while in Study Group 2, it was set at 40% power. During irrigation, patients were instructed to open their mouths and perform lateral mandibular movements to break adhesions, thereby improving vertical and lateral range of motion. (Fig. 3)

### Conventional technique (control group)

**Fig. 4:**
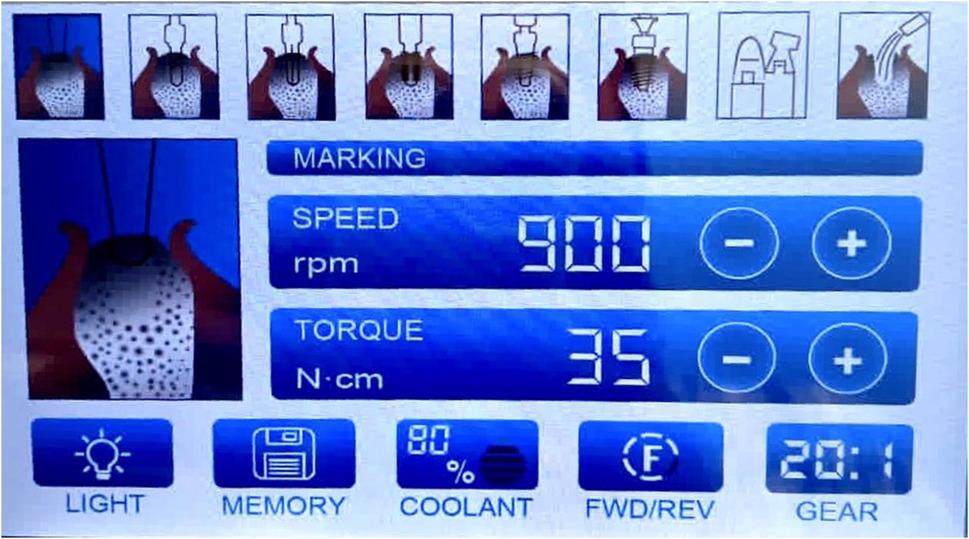
Conventional technique

A total of 250 mL of 0.9% saline solution was injected through the first needle and withdrawn through the second needle using 60-mL syringes. (Fig. 4)

During irrigation, continuous monitoring of fluid outflow from the second needle was maintained. If temporary interruption of outflow occurred, irrigation was immediately stopped, and patency of the output syringe was restored before resuming the procedure. This precaution was taken to minimize the risk of increased intra-articular pressure and potential fluid extravasation.

After completion of irrigation, the second needle was removed, and 1 mL of hyaluronic acid (HA) was injected into the joint. The puncture sites were covered with a spot bandage for 1 h.

### Postoperative phase

Clinical follow-up was scheduled at 1 week, 4 weeks, and 3 months postoperatively to assess pain, maximum mouth opening, and mandibular movements.

Although clinical evaluation was performed at 4 weeks, these data were not included in the final tabulated analysis to avoid redundancy, as the observed trends were consistent with the 1-week and 3-month findings.

### Sample size estimation

Sample size was estimated based on assuming 95% confidence level and 80% study power. The mean improvement in mouth opening after 3 months was 4.2 mm for the conventional arthrocentesis [20] and 7.2 mm for the pumping technique [21]. Based on difference between two independent means using the highest SD = 2 [22] to ensure study power, 9 patients per group are required yielding effect size of 1.5. This was increased to 10 patients to make up for lost follow up cases. Total sample size= number per group × number of groups = 10 × 3 = 30 patients.

Software Sample size was based on Rosner’s method [22] calculated by Gpower 3.0.10 [23]. 

### Statistical analysis of the data

Data were fed to the computer using IBM SPSS software package version 24.0.

Shapiro–Wilk test was used to test of normality of data, the data of different point measurements by two methods was parametric data, the Kolmogorov-Smirnov and Shapiro–Wilk had p value > 0.05.

Qualitative data were described using number and percent. Comparison between different groups regarding categorical variables was tested using Chi-square test.

Quantitative data were described using mean and standard deviation for normally distributed data.

For normally distributed data, comparison between more than two independent population were done using ANOVA-test.

Significance test results are quoted as two-tailed probabilities. Significance of the obtained results was judged at the 5% level.

In addition to p values, effect sizes were calculated for key between-group comparisons using Cohen’s d to quantify the magnitude of differences between study groups.

## Results

### Demographic data and clinical evaluation

The study included thirty patients distributed equally among the three groups. The mean age was 22.4 ± 3.98 years in Group A, 25.6 ± 2.88 years in Group B, and 23.8 ± 3.39 years in Group C. The overall mean age of the sample was approximately 23.9 years. Sex distribution was 2 males & 8 females, 3 males & 7 females, 2 males and 8 females in group A, B and C respectively.

### Pain assessment

**Fig. 5:**
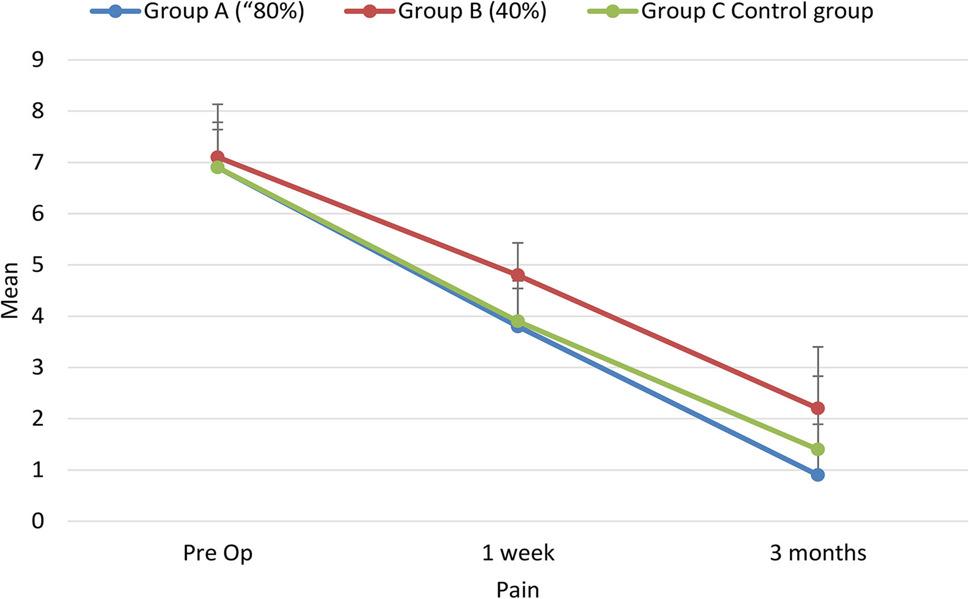
Comparison between the three studied groups regarding Pain at different periods of follow up

Pain scores assessed using the Visual Analog Scale (VAS) showed a statistically significant reduction over time within all groups (*p* < 0.05). Although all groups demonstrated marked postoperative improvement, no statistically significant differences were observed between groups at corresponding time points (*p* > 0.05). These findings are illustrated in Figure 5.

### Maximum Mouth Opening (MMO)

Within-group comparisons revealed a statistically significant increase in MMO across follow-up periods in all groups (ANOVA 1, *p* < 0.05). Between-group comparisons demonstrated no statistically significant differences preoperatively or at 1 week postoperatively (ANOVA 2, *p* = 0.527 and *p* = 0.075, respectively). However, a statistically significant difference was observed at 3 months postoperatively (ANOVA 2, *p* = 0.016). Post hoc analysis showed significant differences between Group A and Group B (*p* = 0.018) and between Group A and Group C (*p* = 0.030), while the difference between Group B and Group C was not statistically significant (*p* = 0.233). Detailed results are presented in Table 1.

**Table 1:** Maximum Mouth Opening (MMO, mm) at different follow-up intervals

Time point	Group A (80%) Mean ± SD	Group B (40%) Mean ± SD	Group C (Control) Mean ± SD	ANOVA (between groups) *p* value
Pre-operative	33.7 ± 2.11	32.8 ± 2.53	33.8 ± 2.53	0.527
1 week	37.5 ± 2.22	35.8 ± 1.87	37.6 ± 1.51	0.075
3 months	41.6 ± 1.26	39.0 ± 1.33	39.7 ± 1.25	0.016*

**Post-hoc comparisons (3 months)**

• Group A vs. B: *p* = 0.018*

• Group A vs. C: *p* = 0.030*

• Group B vs. C: *p* = 0.233

Effect sizes for significant post-hoc comparisons were calculated using Cohen’s d

Within-group comparisons (repeated measures ANOVA)

• Group A: *p* = 0.005*

• Group B: *p* = 0.037*

• Group C: *p *= 0.039*

* Statistically significant at p ≤ 0.05

In addition to statistical significance testing, effect sizes were calculated to assess the magnitude of between-group differences. At the 3-month follow-up, the comparison between Group A and Group B demonstrated a moderate effect size (Cohen’s d = 0.82), while the comparison between Group A and Group C showed a moderate effect size (Cohen’s d = 0.74). The difference between Group B and Group C was associated with a small effect size (Cohen’s d = 0.41). These findings suggest a moderate magnitude of effect; however, clinical relevance should be interpreted cautiously in the absence of predefined MCID thresholds.

(Effect size interpretation based on Cohen’s criteria: small = 0.2, moderate = 0.5, large = 0.8).

### Lateral mandibular movements

All groups demonstrated a statistically significant postoperative improvement in lateral mandibular movements over time (*p* < 0.05). Although Group A showed numerically higher values at the 3-month follow-up, between-group differences did not reach statistical significance at corresponding time points (*p* > 0.05). Results are summarized in Table 2.

**Table 2:** Summary of clinical outcomes of lateral and protrusion Mandibular movements (mean ± SD) for all groups

Variable	Time Point	Group A (80%)	Group B (40%)	Group C
Lateral Movement (mm)	Pre-op	4.50 ± 0.85	4.40 ± 1.26	4.60 ± 1.26
	1 week	6.60 ± 1.07	6.40 ± 1.17	7.10 ± 0.88
	3 months	9.80 ± 1.14	8.10 ± 1.20	9.20 ± 0.92
Protrusion (mm)	Pre-op	5.00 ± 0.82	4.70 ± 0.67	4.90 ± 0.88
	1 week	7.10 ± 0.99	6.70 ± 0.95	7.10 ± 0.88
	3 months	8.90 ± 0.74	8.70 ± 0.95	8.90 ± 0.74

### Protrusion movements

Protrusive mandibular movements increased significantly over time within all study groups (*p* < 0.05). No statistically significant differences were detected between groups at any follow-up interval (*p* > 0.05), as shown in Table (2).

### Procedure time

**Fig. 6:**
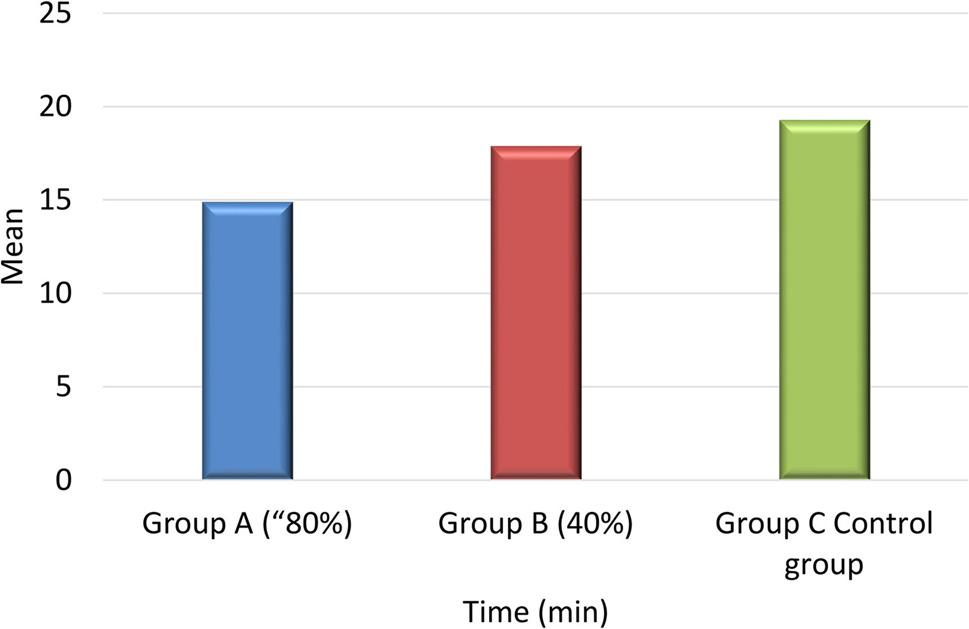
Comparison of mean procedure time (minutes) among the three study groups

A statistically significant difference in procedure time was observed among the three groups (one-way ANOVA, *p* = 0.001). Post-hoc analysis demonstrated that Group A had a significantly shorter procedure time compared to Group B (*p* = 0.013) and Group C (*p* = 0.001). Additionally, a statistically significant difference was observed between Group B and Group C (*p* = 0.042) Figure 6.

Minor postoperative swelling was observed in a small number of patients in both the implant motor–assisted and conventional arthrocentesis groups. The swelling was transient, mild in intensity, and resolved spontaneously within a few hours without the need for additional intervention. No major complications such as persistent extravasation, hematoma, facial nerve injury, or infection were encountered.

## Discussion

This randomized controlled trial compared two lavage delivery methods for TMJ arthrocentesis in patients with disc displacement with reduction (DDwR). All treatment groups demonstrated significant improvement in maximum mouth opening (MMO), pain intensity, and mandibular movements over the follow-up period. The high-power motor-assisted arthrocentesis technique was associated with a significantly shorter procedure time compared with the conventional technique, while functional outcomes were broadly comparable between groups.

Regarding the primary outcome, MMO improved significantly over time in all study groups. These improvements across all groups may be attributed to lavage-mediated reduction of intra-articular inflammatory mediators, disruption of minor adhesions, and improvement of intra-articular biomechanics. Although the motor-assisted groups showed slightly greater numerical improvements, intergroup differences were not consistently statistically significant. At the 3-month follow-up, a statistically significant difference in MMO was observed between the high-power motor-assisted group and the conventional technique; however, the absolute difference was approximately 1.9 mm. This magnitude falls below commonly reported minimal clinically important difference (MCID) thresholds for MMO following TMJ arthrocentesis, which are generally estimated at 5 mm or greater. Accordingly, despite achieving statistical significance, this difference is unlikely to represent a clinically meaningful improvement and should be interpreted cautiously when considering therapeutic superiority.

Pain intensity also decreased significantly over time in all groups. This improvement may be explained by the fundamental therapeutic mechanisms of arthrocentesis, including dilution and removal of intra-articular inflammatory mediators, reduction of negative intra-articular pressure, improvement of synovial fluid circulation, and disruption of minor adhesions within the superior joint compartment. Although numerically greater pain reduction was observed in the motor-assisted groups, these differences did not reach statistical significance when compared with the conventional technique, suggesting that pain relief is largely attributable to the arthrocentesis procedure itself rather than the specific irrigation delivery method.

Regarding operative time, a significant difference was observed, with the dental implant motor irrigation pump technique using 80% power (Group A) showing a notably shorter mean operative time compared with the other two groups. This may be attributed to the controlled and continuous flow provided by the implant motor pump, which facilitates joint lavage and improves procedural efficiency. To the best of our knowledge, it is the first study to evaluate and compare the operation time of TMJ arthrocentesis using implant motor irrigation pump versus the conventional technique.

As for protrusive mandibular movements, all groups exhibited a statistically significant improvement during the follow-up period. This improvement may be attributed to the reduction of intra-articular inflammation, decreased joint friction, and improved condylar translation following joint lavage. Although intergroup differences were not statistically significant, Group A consistently demonstrated slightly higher mean values, suggesting a possible trend toward improved functional recovery.

These results are in agreement with those of Grossmann and Poluha, who reported superior functional recovery when a constant-pressure irrigation system was used during arthrocentesis [24]. The results of this study are also consistent with several previous reports demonstrating the effectiveness of arthrocentesis in improving mouth opening and reducing pain in patients with TMJ internal derangement, where significant postoperative increases in maximal mouth opening and decreases in pain scores were observed following arthrocentesis procedures [25]. The present results support these observations while emphasizing that the primary added benefit of motor-assisted irrigation lies in procedural efficiency rather than clear functional superiority.

The superior performance of the implant motor irrigation pump technique may be explained by its ability to provide consistent pressure and more complete lavage, reducing residual debris and inflammatory substances within the joint. However, the procedural comparison presented here does not replace conservative care principles but rather examines technical optimization within an already indicated minimally invasive intervention.

The findings of the present study should be interpreted within the broader biopsychosocial context of DDwR. DDwR is widely recognized as a condition with a generally favorable natural history, where symptoms such as pain and functional limitation may improve over time through reassurance, behavioral modification, and conservative self-management, even in the absence of invasive intervention. Consequently, the functional improvements observed in this study—including reductions in pain intensity and increases in mandibular mobility—cannot be attributed solely to the arthrocentesis procedure or the irrigation delivery method. Rather, these changes likely reflect the combined influence of natural symptom fluctuation, conservative adjunctive measures (including splint therapy), and the minimally invasive intervention itself. From a contemporary biopsychosocial perspective, DDwR should not be conceptualized as a purely mechanical disorder requiring anatomical correction, but rather as a multifactorial condition in which biological, behavioral, and psychosocial factors interact to influence symptom persistence and recovery. In this context, the present study does not propose lavage optimization as a standalone solution, but instead evaluates technical refinement within an already indicated, conservative-to-minimally invasive care pathway. In addition, the mandatory use of anterior repositioning splint therapy and intra-articular hyaluronic acid injection in all study groups represents an important co-intervention effect. Therefore, the observed improvements cannot be attributed solely to the irrigation delivery method, but rather to the combined therapeutic protocol.

Furthermore, although DDwR is often considered a condition with a favorable natural course, the present study specifically included patients with persistent symptoms who did not respond adequately to initial conservative management, thereby justifying the use of minimally invasive intervention in this selected subgroup.

The selection of 40% and 80% power settings for the dental implant motor irrigation pump was based on clinical considerations balancing irrigation efficiency and patient safety. The lower setting (40%) was chosen to represent a conservative irrigation pressure used to minimize the risk of excessive intra-articular pressure, while the higher setting (80%) was selected to evaluate the potential benefits of increased and sustained irrigation flow on joint lavage and operative time.

Minor transient swelling was observed in both motor-assisted and conventional groups, which may be attributed to limited fluid extravasation commonly associated with TMJ arthrocentesis. Importantly, no major adverse events were recorded. Continuous intraoperative monitoring of outflow and immediate interruption of irrigation when necessary likely contributed to minimizing pressure-related complications. These findings suggest that, with appropriate technical precautions, motor-assisted arthrocentesis can be performed safely.

The use of an anterior repositioning splint in conjunction with arthrocentesis was intentionally incorporated into the treatment protocol. Splint therapy may enhance treatment efficacy by reducing joint loading, stabilizing mandibular position, decreasing parafunctional activity, and creating a more favorable biomechanical environment for joint healing. Previous studies have demonstrated that combining arthrocentesis with repositioning splint therapy results in greater improvement in pain relief and mandibular function compared to arthrocentesis alone. Navaneetham et al. (2023) reported favorable outcomes using this combined approach, supporting its clinical relevance [26]. In the present study, splint therapy was applied uniformly to all patients to eliminate its influence as a confounding variable, thereby allowing a more accurate comparison between the tested arthrocentesis techniques.

Although the total duration of anterior repositioning splint therapy was standardized to three months for all patients, the preoperative splint therapy period was not strictly standardized. The proportion of splint use before versus after arthrocentesis may have varied among participants according to individual clinical response and scheduling factors. This variability may have influenced baseline symptoms and treatment response and represents a potential confounding factor. Future studies should standardize the preoperative splint therapy duration or control for its timing to better isolate the procedural effect.

It is important to emphasize that the present study does not aim to promote a purely mechanical interpretation of temporomandibular disorders (TMDs). Rather, it evaluates two procedural variants of a minimally invasive intervention within a specific subgroup of patients diagnosed with disc displacement with reduction. Contemporary TMD management frameworks, including INfORM/IADR key points, advocate a biopsychosocial approach and emphasize reversible, conservative interventions. In this context, arthrocentesis remains an accepted minimally invasive option for carefully selected patients when conservative measures are insufficient.

A further important limitation relates to the absence of a formal DC/TMD Axis II psychosocial assessment. Psychosocial factors that may influence pain perception, disability, and treatment response were therefore not systematically evaluated. This may contribute to patient heterogeneity and could have influenced the observed clinical outcomes. Future studies should incorporate standardized Axis II assessment to enable more comprehensive interpretation of treatment response within a biopsychosocial framework.

It is important to clarify that the therapeutic objective in the present study was symptomatic and functional improvement rather than anatomical disc “recapture.” Structural disc position does not necessarily correlate with pain or functional limitation, and clinical success was therefore defined by pain reduction and improvement in mandibular movement parameters.

Although pain intensity was quantified using a visual analog scale, the absence of validated patient-reported outcome measures (PROMs), such as pain-related disability or quality-of-life instruments, limits the ability to fully contextualize the clinical relevance of the observed functional improvements. Without these measures, it is not possible to determine the extent to which statistically significant changes translated into meaningful patient-perceived benefit.

The relatively small sample size may have limited the statistical power of the study to detect modest intergroup differences. Accordingly, both statistically significant and non-significant findings should be interpreted with caution, considering the potential risk of type I and type II errors.

In addition, the relatively short follow-up period may have limited the assessment of long-term outcomes. The 3-month follow-up period was chosen to assess early clinical and functional outcomes following TMJ arthrocentesis, during which most symptomatic improvements are expected to occur. According to Alpaslan et al., no significant differences were observed when comparing shorter and longer follow-up outcomes after TMJ arthrocentesis [27]. A longer follow-up was not feasible due to logistical constraints and the predefined study timeline. Nevertheless, the authors acknowledge that longer follow-up durations are essential to assess the long-term stability and durability of treatment outcomes. Future prospective studies with extended follow-up periods are planned to further investigate the long-term clinical performance of the implant motor irrigation pump technique.

The technique also requires a specialized implant motor, adding extra cost and potentially limiting its broader clinical use.

A further important limitation of this study is that trial registration was completed retrospectively, after participant recruitment had concluded. This may reduce methodological transparency and introduces a potential risk of selective reporting bias in randomized clinical trials. Although the study protocol, outcome definitions, and statistical analysis plan were pre-specified and remained unchanged throughout recruitment and data analysis, the lack of prospective registration should be carefully considered when interpreting the findings.

The sample size of this study was calculated based on the primary outcome (maximum mouth opening) and was not powered to detect between-group differences in secondary outcomes such as pain intensity, lateral excursions, and protrusion. Therefore, non-significant findings for these secondary outcomes should not be interpreted as evidence of equivalence between the compared techniques, and insufficient statistical power may have influenced these results.

Occlusal variations were not formally stratified in the analysis, which may represent a minor source of measurement variability. However, consistent reference points were maintained across all follow-up assessments to minimize this effect.

MRI interpretation was performed by a single experienced radiologist, and inter-observer reliability was therefore not assessed. Although predefined radiologic criteria based on established TMJ MRI standards were applied and the radiologist was calibrated prior to study initiation, the absence of formal intra- or inter-observer reliability testing represents a methodological limitation and may affect the reproducibility of the radiological diagnosis.

Although MMO was selected as the primary endpoint due to its objective and reproducible nature, the absence of a predefined minimal clinically important difference (MCID) limits the interpretation of the clinical magnitude of change. Future studies incorporating MCID thresholds and patient-centered outcome measures are warranted.

The absence of assessor blinding represents a potential source of measurement bias, particularly for clinically assessed outcomes such as mouth opening and mandibular movements. Although repeated measurements and high intra- and inter-examiner reliability were achieved, the possibility of observer bias cannot be excluded.

One limitation of the present study is that the exact duration of disc displacement was not assessed; however, treatment protocols were standardized across all study groups to minimize its potential impact on the outcomes.

Regarding external validity, the study was conducted on real-world clinical cases at a university hospital, including patients of both genders and a relevant age range. This enhances the generalizability of the findings to similar clinical settings and populations.

## Conclusion

Both conventional and dental implant motor–assisted arthrocentesis resulted in significant functional improvement in patients with DDwR. The motor-assisted technique was associated with shorter operative time, while functional outcomes were largely comparable between techniques. However, given the small sample size, lack of assessor blinding, and the presence of co-interventions, these findings should be interpreted with caution. Further well-designed, adequately powered randomized trials are required to confirm these results and clarify the independent effect of irrigation delivery methods.

## Supplementary Information

Supplementary Material 1.

## Data Availability

The datasets used and/or analyzed during the current study are available from the corresponding author upon reasonable request.

## Abbreviations

TMJ: Temporomandibular Joint
TMJID: Temporomandibular Joint Internal Derangement
TMDs: Temporomandibular Disorders
MRI: Magnetic Resonance Imaging
MMO: Maximum Mouth Opening
SD: Standard Deviation
VAS: Visual Analogue Scale
DDWR: Disc Displacement with Reduction
